# Management of Infants with Brief Resolved Unexplained Events (BRUE) and Apparent Life-Threatening Events (ALTE): A RAND/UCLA Appropriateness Approach

**DOI:** 10.3390/life11020171

**Published:** 2021-02-22

**Authors:** Giovanni Prezioso, Serafina Perrone, Giacomo Biasucci, Giovanna Pisi, Valentina Fainardi, Caterina Strisciuglio, Francesco Nonnis Marzano, Sabrina Moretti, Francesco Pisani, Bertrand Tchana, Alberto Argentiero, Cosimo Neglia, Carlo Caffarelli, Patrizia Bertolini, Maria Teresa Bersini, Andrea Canali, Emanuele Voccia, Antonella Squarcia, Tullio Ghi, Carla Verrotti, Tiziana Frusca, Rossana Cecchi, Giovanna Giordano, Filomena Colasanti, Ilenia Roccia, Paola Palanza, Susanna Esposito

**Affiliations:** 1 Pediatric Clinic, Department of Medicine and Surgery, University of Parma, 43126 Parma, Italy; gprezioso@ao.pr.it (G.P.); gpisi@ao.pr.it (G.P.); valentina.fainardi@gmail.com (V.F.); francesco.pisani@unipr.it (F.P.); alberto.argentiero@unipr.it (A.A.); negliamino@gmail.com (C.N.); carlo.caffarelli@unipr.it (C.C.); 2 Unit of Neonatology, Department of Medicine and Surgery, University of Parma, 43126 Parma, Italy; serafina.perrone@unipr.it (S.P.); smoretti@ao.pr.it (S.M.); fcolasanti@ao.pr.it (F.C.); iroccia@unipr.it (I.R.); 3 Pediatrics and Neonatology Unit, Guglielmo da Saliceto Hospital, 29122 Piacenza, Italy; g.biasucci@ausl.pc.it; 4 Department of Woman, Child and General and Specialistic Surgery, University of Campania “Luigi Vanvitelli”, 80138 Naples, Italy; caterinastrisciuglio@hotmail.it; 5 Department of Chemistry, Life Sciences and Environmental Sustainability, University of Parma, 43126 Parma, Italy; francesco.nonnismarzano@unipr.it; 6 Cardiology Unit, Parma Hospital, 43126 Parma, Italy; btchana@ao.pr.it; 7 Oncohematology Unit, Parma Hospital, 43126 Parma, Italy; pbertolini@ao.pr.it; 8 Primary Care Pediatrics, AUSL of Parma, 43126 Parma, Italy; gsfmt@libero.it (M.T.B.); andrea.canali@medici.progetto-sole.it (A.C.); e.voccia@libero.it (E.V.); 9 Unit of Neuropsychiatry of Children and Adolescents, AUSL Parma, 43126 Parma, Italy; asquarcia@ausl.pr.it; 10 Obstetrics and Gynaecology Unit, Department of Medicine and Surgery, University of Parma, 43126 Parma, Italy; tullio.ghi@unipr.it (T.G.); tiziana.frusca@unipr.it (T.F.); 11 Woman Health Unit, AUSL Parma, 43126 Parma, Italy; cverrotti@ausl.pr.it; 12 Legal Medicine Section, Department of Medicine and Surgery, University of Parma, 43126 Parma, Italy; rossana.cecchi@unipr.it; 13 Pathology Unit, Department of Medicine and Surgery, University of Parma, 43126 Parma, Italy; giovanna.giordano@unipr.it; 14 Unit of Neuroscience, Department of Medicine and Surgery, University of Parma, 43126 Parma, Italy; paola.palanza@unipr.it

**Keywords:** ALTE, BRUE, pediatric emergency, RAND/UCLA appropriateness method, SIDS

## Abstract

Unexpected events of breath, tone, and skin color change in infants are a cause of considerable distress to the caregiver and there is still debate on their appropriate management. The aim of this study is to survey the trend in prevention, decision-making, and management of brief resolved unexplained events (BRUE)/apparent life-threatening events (ALTE) and to develop a shared protocol among hospitals and primary care pediatricians regarding hospital admission criteria, work-up and post-discharge monitoring of patients with BRUE/ALTE. For the study purpose, a panel of 54 experts was selected to achieve consensus using the RAND/UCLA appropriateness method. Twelve scenarios were developed: one addressed to primary prevention of ALTE and BRUE, and 11 focused on hospital management of BRUE and ALTE. For each scenario, participants were asked to rank each option from ‘1’ (extremely inappropriate) to ‘9’ (extremely appropriate). Results derived from panel meeting and discussion showed several points of agreement but also disagreement with different opinion emerged and the need of focused education on some areas. However, by combining previous recommendations with expert opinion, the application of the RAND/UCLA appropriateness permitted us to drive pediatricians to reasoned and informed decisions in term of evaluation, treatment and follow-up of infants with BRUE/ALTE, reducing inappropriate exams and hospitalisation and highlighting priorities for educational interventions.

## 1. Introduction

Unexpected events of breath, tone, and skin color change in infants are a cause of considerable distress to the caregiver, often pushed to seek emergency medical care. The clinical management of infants with these signs and symptoms may result troublesome, both for identifying a possible cause and for reducing the parents’ anxiety. Before 1986, the definition near-miss sudden infant death syndrome (SIDS) was commonly used to define episodes of an infant found apparently dead and requiring stimulation or resuscitation manoeuvres. In that year, an American expert panel introduced the acronymous ALTE (apparent life-threatening events) for episodes frightening to the observer and characterised by some combination of apnoea, color change, marked change in muscle tone, choking, or gagging in patients younger than 12 months of age, also stating that these events should not be considered as closely associated with SIDS [1].

However, the definition of ALTE entailed a subjective assessment of symptoms by the witness, frequently unable to distinguish benign, physiological conditions from minor or severe events. Furthermore, the attribute ‘life-threatening’ generated anxiety in caregivers and induced clinicians to perform inappropriate diagnostic testing in often well appearing patients. In 2016, the American Academy of Pediatrics (AAP) Sub-Committee on Apparent Life Threatening Events proposed to define as “brief resolved unexplained events” (BRUE) any brief, transient, resolved events in children less than 12 months of age with no explainable cause after careful medical examination [2]. The intent was both to leave the clinician the characterization of the episode and to reduce the caregiver’s perception of severity. According to the authors’ evidence-based practice, the term BRUE should be used when the observer describes at least one among the following signs: cyanosis or pallor, absent, decreased or irregular breathing, marked change in tone, altered responsiveness. The infant should also present to the emergency room (ER) in good clinical condition. In any case, BRUE might hide an undiagnosed serious condition or an increased probability of recurrence; therefore, an accurate stratification from low to high risk is also recommended by the American guidelines, which then provided key action statements for the low-risk cases [2]. In 2017, the Italian Society of Pediatrics updated its own guidelines and accepted the new acronymous BRUE for idiopathic and minor episodes, while keeping the term ALTE for severe/higher risk cases, namely related to patient instability, prematurity (<32 weeks of gestational age and <43 weeks of post-conceptional age), age below 2 months, need for resuscitation manoeuvres, recurrence, and poor family compliance [3]. The Italian Committee also focused on diagnostic work-up, hospital management and clinical follow-up of ALTE, providing a valid support for physicians, who routinely deal with this emerging condition [3].

Indeed, the estimated incidence of ALTE varies among studies from 0.6 to 2.46 per 1000 live births, constituting up to the 0.8% of total pediatric emergency visits for children below 12 months of age [4]. The SIDS prevention campaigns directed to new parents together with a heightened awareness among pediatricians are probably contributing to increase ALTE incidence. Though some years have passed since the Italian guideline publication, the appropriate management of ALTE is still debated; this may be also due to several recommendations published thereafter, though limited by a very low level of evidence. The aim of this study is to survey the trend in prevention, decision-making and management of BRUE/ALTE and to develop a shared protocol among hospital and primary care pediatricians regarding hospital admission criteria, work-up, and post-discharge monitoring of patients with BRUE/ALTE.

## 2. Materials and Methods

### 2.1. RAND/UCLA Appropriateness Method

For the study purpose, a panel of experts was selected to achieve consensus using the Research and Development Corporation (RAND) and the University of California – Los Angeles (UCLA) appropriateness method [5], a system that combines scientific evidence with experts’ opinion, especially when current evidence for conditions and procedures is insufficient. The RAND/UCLA appropriateness method was developed by RAND and the UCLA in 1980s with the aim of determining the overuse or underuse of medical and surgical procedures through their level of appropriateness. The concept of appropriateness refers to those interventions in which expected health benefits exceed the expected negative consequences by a sufficiently wide margin such that the procedure is worth doing, exclusive of costs [6]. The RAND/UCLA method can be considered a variant of the Delphi technique, as it involves a group of experts/specialists reading a detailed literature review, then answering anonymously to a questionnaire, followed by an open discussion of the survey and by a possible second round questionnaire to minimize possible disagreements [7]. Thus, each indication is rated as ‘appropriate’, ‘inappropriate’, or ‘uncertain’. In the previous decades, the RAND/UCLA method was employed in several countries to evaluate appropriateness of medical procedures, such as diagnosis and management of glaucoma [8], use of intravenous or urinary catheters [9], and imaging procedures [10,11,12]. However, in the field of pediatrics this method is still underemployed.

### 2.2. Literature Search

Two reviewers (GPr and AA) independently searched for experimental studies, reviews, systematic reviews, metanalysis, guidelines using the MEDLINE database (search strategy: [(ALTE) OR (BRUE) and (management)]) published from 1990 to date. Only English language articles were selected. Secondly, the search was completed by a manual review of articles and bibliographies. The selected papers were then provided to the panellists to ensure them an equal and appropriate body of evidence.

### 2.3. Questionnaire Development

Using the above-mentioned American and Italian guidelines, 12 scenarios were developed: one addressed to primary prevention of ALTE and BRUE, and 11 focused on hospital management of BRUE and ALTE. For each scenario, a series of options were provided and panellists were asked to rank each option from ‘1’ (extremely inappropriate) to ‘9’ (extremely appropriate), as indicated by the RAND/UCLA users’ manual [6]. 

### 2.4. Panel Selection

After curriculum vitae evaluation, a multidisciplinary group of 54 Italian specialists in the field of neonatology, general pediatrics and pediatrics subspecialties, gynaecology/obstetrics, genetics, forensic medicine, and pathology was randomly selected among those working in Parma province, an area of Emilia-Romagna Region in Italy with 453,317 inhabitants. All panellists were recruited via phone and email contact and, after acceptance, each one of them was provided with a panel document including the literature review, definitions and instructions, and an online questionnaire.

### 2.5. First Round and Data Analysis

In step one, the questionnaire was sent to the participants using the online survey application ‘Google Forms’. Participants were given two weeks to review all documents and anonymously rank the appropriateness of each scenario and indication from ‘1’ to ‘9’ (ranging from extremely not appropriate to extremely appropriate), as aforementioned. Grades from ‘1’ to ‘3’ were considered inappropriate, grades from ‘4’ to ‘6’ were considered indeterminate or equivocal, and grades from ‘7’ to ‘9’ were considered appropriate. Responses were collected and sent to an independent statistician who synthesized results. 

Aggregate results were reported as frequencies and medians. The median (mdn) panel ratings were classified into three levels of appropriateness (appropriate: between ‘7’ and ‘9’, without disagreement; uncertain: between ‘4’ and ‘6’ or any median with disagreement; inappropriate: between ‘1’ and ‘3’ with agreement). Agreement was reached in case of at least 75% of respondents ranking within the same level of appropriateness. A 70% or higher level of agreement was still considered adequate after second round in case of crucial indications supported by previous guidelines. 

The data analysis was performed with the STATA® Statistical Software (Release 11 College Station, TX, College Station, Texas, USA). For each question, the answers were divided into three groups of appropriateness, each encompassing three grades of appropriateness (Group 0 included grades 1 to 3; group 2 including grades 3 to 6; group 3 including grades 7 to 9). The mean value with 95% confidence interval was then calculated. Microsoft Excel® was used for graphic data processing.

### 2.6. Consensus Meeting and Second Round: Definition of Disagreement/Agreement

Results of the survey were discussed in a web meeting in 16–17 November 2020, where the collective ranking of scenarios and indications was showed. Clarifications, adaptations, and refinements of the indications and appropriateness ratings were made; thus, participants were asked to re-rank the scenarios in a second round during the following two weeks. The second round results were considered definitive and are shown in graphical form in Supplementary Material.

### 2.7. Protocol

**Figure 1:**
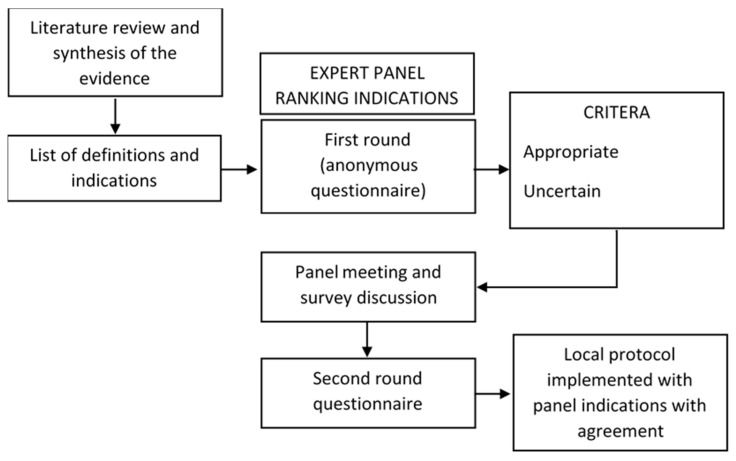
RAND/UCLA appropriateness approach on management of infants with brief resolved unexplained events (BRUE)/apparent life-threatening events (ALTE).

The protocol resulting from our survey combined guidelines recommendations, in particular those supported by the higher level of evidence [2,3], and the more appropriate and agreed indications approved by the panel. In case of clear conflict between guidelines and panel indications, the moderator (SE) discussed with participants about the recommendations most conform to local needs. Figure 1 summarizes the study approach.

## 3. Results

### 3.1. Study Participants

Among the 54 specialists selected in the panel, 14 worked in a hospital setting and 40 in primary care (44 were employed in urban and 10 in rural areas). The age distribution of the participants showed a prevalence of thirties (38 out of 54), followed by clinicians aged 40–49 years (n = 6) and over 50 (n = 10). No drop-out occurred in the second round.

### 3.2. Scenario 1—Prevention of ALTE/BRUE

The panel considered eight indications addressed to primary prevention of ALTE/BRUE. Agreement was obtained for: recommending parents to place the infant to sleep on his back (100% appropriate; mdn: 9) using semirigid mattress (90.9% appropriate; mdn: 9), letting the baby sleep in parents’ bedroom for the first six months (81.8% appropriate; mdn: 8), confirming the absence of risks associated with the use of pacifiers at sleep onset (100% appropriate; mdn: 9), promoting breastfeeding and vaccinations (100% appropriate; mdn: 9), advising against exposure to second-hand smoke (100% appropriate; mdn: 9). Lack of consensus resulted for not suggesting sideways position after feeding (72.7% inappropriate; mdn: 3) and avoiding use of pillow in the baby’s bed (63.6% appropriate; mdn: 3), although the latter indication was discouraged by the AAP [2].

### 3.3. Scenario 2—Emergency Room (ER) Evaluation

The participants ranked seven indications regarding the necessity of a prompt ER medical evaluation on the basis of risk factors presented by a patient with BRUE/ALTE (as discussed in the American [2] and Italian [3] guidelines). Five cases obtained agreement: first episode lasting less than one minute in age-corrected premature infant <43 weeks (90.9% appropriate; mdn: 9), recurrent episodes of brief (<1 min) duration in an infant >2 months old (81.8% appropriate; mdn: 9); recurrent episodes lasting more than one minute in age-corrected premature infant <43 weeks (100% appropriate; mdn: 9); any first episode lasted <1 min in a context of poor family compliance (100% appropriate; mdn: 9); any episode with familial history of SIDS (100% appropriate; mdn: 9). Lack of consensus derived from the indication for ER evaluation in a first episode of brief duration in infants >2 months old, irrespective of other risk factors (45.5% appropriate, 27.3% uncertain, 27.3% inappropriate; mdn: 5), as well as in the case of irregular breathing lasted >1 min without color or muscle tone change in a patient below 2 months of age (27.3% appropriate, 36.4% uncertain, 36.4% inappropriate; mdn: 4).

### 3.4. Scenario 3—Hospital Admission

With regard to the Italian Society of Pediatrics guidelines, six indications for hospital admission were submitted to participants, all of which received full agreement: unstable patient (100% appropriate; mdn: 9), need for resuscitation maneuvers (90.9% appropriate; mdn: 9), age less than two months (100% appropriate; mdn: 9), age corrected premature infant <43 weeks (100% appropriate; mdn: 9), recurrent episodes (100% appropriate; mdn: 9), and poor family compliance (100% appropriate; mdn: 9).

### 3.5. Scenario 4—Management of Patients Who Do Not Need Hospitalization

For all those cases of BRUE not requiring hospitalization in accordance with the aforementioned guidelines and indications derived from the first round consensus, the panel evaluated five indications for clinical management. However, second round obtained consensus only for performing an electrocardiogram (81.9% appropriate; mdn: 9). Despite a high level of appropriateness, performing routine blood tests (72.7% appropriate; mdn: 7), neurological examination (72.7% appropriate; mdn: 9), and carring out routine chest X-ray in any case (72.7% inappropriate; mdn: 3) did not obtain an adequate level of agreement. No consensus was achieved for the time of observation, and two-to-four hours threshold was considered appropriate by 54.5% of participants, versus 27.3% who considered that time inappropriate and 18.2% uncertain (mdn: 7).

### 3.6. Scenario 5—Management of BRUE/ALTE Cases Associated with Gastrointestinal Symptoms

With regard to the scenario of a patient with BRUE/ALTE presenting with regurgitations and/or other gastrointestinal symptoms, the panel was required to examine and rank seven indications. Four of them were rated with agreement. The participants considered appropriate the use of pH-impedance as diagnostic test for gastro-esophageal reflux disease (GERD) when extra-esophageal symptoms suggestive of GERD occur (i.e., recurrent airways inflammation, Sandifer syndrome) (81.8% appropriate; mdn: 9), rather than in any case of suspected GERD on the basis of esophageal symptoms only (36.4% inappropriate, 36.4% uncertain, 27.3% appropriate; mdn: 5). On the contrary, esophagogastroduodenoscopy (EGDS) was considered appropriate when typical symptoms (i.e., hematemesis, reflux after the first year of life, poor growth, feeding difficulties, iron deficiency anemia) occur (81.8% appropriate; mdn: 9), rather than in any case of suspected GERD (45.5% inappropriate, 18.2% uncertain, 36.4% appropriate; mdn: 5). Performing an esophagogastric ultrasound scan was judged as inappropriate for the 36.6% of participants (mdn: 3). No consensus was achieved about the indication to start a trial with alginate in troublesome regurgitating infants before GERD investigation (45.5% inappropriate, 20.4% uncertain, 45.4% appropriate; mdn: 5), whereas a trial with a pump inhibitor was ranked inappropriate (63.6% inappropriate; mdn: 3).

### 3.7. Scenario 6. First-Line Tests to Perform in Hospitalized Patients with ALTE

Among the nine options presented to the participants, eight reached agreement: complete blood count with formula (100% appropriate; mdn: 9), electrolytes and blood gas analysis (100% appropriate; mdn: 9), glycemia (100% appropriate; mdn: 9), C-reactive protein (100% appropriate; mdn: 9), renal and hepatic function tests (81.8% appropriate; mdn: 9), electrocardiogram (ECG) (90% appropriate; mdn: 9), and cardiorespiratory monitoring for 24 h or more (90.9% appropriate; mdn: 9). Urinalysis just reached an under the limit rate for appropriateness (72.7% appropriate; mdn: 9). Routine chest X-ray did not obtain consensus (27.3% inappropriate, 36.4% uncertain, 36.4% appropriate; mdn: 4).

### 3.8. Scenario 7—Investigations for Infectious Disease in BRUE/ALTE Patients

The majority of participants considered appropriate the indication of performing investigations for infection only if a patient with ALTE presents with a suspected respiratory infection, despite the inadequate rate of consensus (63.6% appropriate; mdn: 9). In particular, the search for *Bordetella pertussis* in patients who received ≤1 hexavalent vaccination dose was ranked appropriate with consensus (90.9% appropriate; mdn: 8). Indeterminate agreement was obtained for testing for respiratory syncytial virus only in epidemic seasons (63.6% appropriate; mdn: 9).

### 3.9. Scenario 8—Indication to Polysomnography

The panel confirmed the previously published recommendations for polysomnography [13], i.e., a history of sleep breathing problems (81.8% appropriate; mdn: 9) or suspected central apnea (81.8% appropriate; mdn: 9).

### 3.10. Scenario 9—When to Suspect a Metabolic Disease 

Among all conditions that might be associated with metabolic disorders, some indications were extracted from the literature and clinical experience and presented to participants. Consensus was reached for a positive family history of SIDS (81.8% appropriate; mdn: 8) and the association of vomiting with neurological symptoms (81.8% appropriate; mdn: 9). On the other hand, a history of frequent regurgitation achieved a final disagreement for performing further metabolic work-up (36.4% inappropriate, 54.5% uncertain, 9.1 appropriate; mdn: 4).

### 3.11. Scenario 10—Work-Up in Case of Suspected Metabolic Disease

Five out of six tests ranked by participants were considered as appropriate to screen cases of ALTE likely depending to a congenital error of metabolism: blood gas analysis (90.9% appropriate; mdn: 9), blood ammonia (100% appropriate; mdn: 9), urinary organic acids and plasma amino acids (90.9% appropriate; mdn: 9), glycemia (100% appropriate; mdn: 9), ketonemia (90.9% appropriate; mdn: 9). The indication for array comparative genomic hybridization (aCGH) reached an undetermined agreement (18.2% inappropriate, 36.4% uncertain, 45.4% appropriate; mdn: 6).

### 3.12. Scenario 11—Hospital Pulse Oximetry Monitoring

The application of a cardiorespiratory monitor was considered appropriate in any case of BRUE or ALTE, but a higher level of agreement was reached for applying it only in ALTEs (72.7% versus 81.8% appropriate; mdn: 7 vs. 8). In high-risk cases (ALTE according to the Italian guidelines), consensus was achieved for extending pulse oximetry monitoring for at least 24 h (81.8% appropriate; mdn: 9), whereas neither a 4-h-length nor a 12-h-length threshold for monitoring low risk cases (BRUE) reached agreement (respectively, 54.5% appropriate; mdn: 7; and 54.5% appropriate; mdn: 7).

### 3.13. Scenario 12—Home Cardiorespiratory Monitoring

With respect to the possibility of home monitoring prescription, participants ranked appropriate the indication for pulse oximetry for patients with recurrent ALTEs (81.8% appropriate; mdn: 9), ALTE in premature and postconceptional age <43 weeks (77.8% appropriate; mdn: 9), and family history of SIDS (81.8% appropriate; mdn: 9). Appropriateness of a 12-week-length monitoring was rated with disagreement (18.2% inappropriate, 27.3% uncertain, 54.5% appropriate; mdn: 7).

### 3.14. Protocol

#### 3.14.1. Primary Prevention

Regarding primary prevention, we highlighted that neonatologists and pediatricians must emphasize the importance of breastfeeding, vaccinations, supine position during sleep, use of firm mattresses without soft objects on the sleep surface or covering the baby. The use of pacifiers at the sleep onset may be suggested, especially after the first month of life and breastfeeding stabilization, without forcing the baby in case of refusal. The infant’s bed should be kept in the parents’ room at least for the first six months. Moreover, recommending to avoid first-hand and second-hand smoke exposure is paramount during pregnancy and after birth. Local educational campaigns targeted at parents on SIDS and ALTE/BRUE should be promoted with the use of brochures and posters.

#### 3.14.2. Emergency Management of BRUE and ALTE

Any unexpected events of breath, tone, and skin color change in infants may require a medical consultation. However, a prompt ER evaluation should be recommended in case of first episode of suspected BRUE or ALTE (see definitions in Table 1) in preterm infants below 32 weeks of gestational age and a postconceptional age lower than 45 weeks, any event in at-term infants below 60 days of life, any high-risk (ALTE) episode, recurrent episodes of BRUE/ALTE, known background of poor family compliance or social issues. The first and most important step of ER assessment is the history collection, since the patient usually comes to doctor’s attention when critical symptoms have passed. A careful description of the event should be collected from the direct witness, the caregiver, and the one who provided first aid. In order to distinguish between real data and emphasized information due to the caregiver’s anxiety, a two-time collection is suggested, with a second interview carried out after a few hours of observation. Secondly, information on pregnancy, birth, past medical history, and family history must be collected to assess risk factors, with specific attention to patient’s conditions during the last 24 h prior to the event (Table 2).

An extensive physical examination is the other key point of ER and risk assessment. General clinical conditions of the baby, vital parameters at first evaluation, and cardiopulmonary and neurologic conditions have to be evaluated together with any sign of gastrointestinal involvement, infection, abuse, and dysmorphism (Table 3). Once all data have been collected, a risk stratification could drive clinicians for hospitalization and further evaluations. A high-risk patient is characterized by at least one of the following features: age < 60 days, preterm with gestational age < 32 weeks and post-conceptional age < 45 weeks, recurrent episodes, resuscitation maneuvers required during the episode, medical history and/or physical examination.

Cardiorespiratory monitoring in ER is indicated in high-risk patients, while low-risk patients should undergo at least two hours of observations, possibly with pulse oximetry monitoring. In both cases, similarly to the history collection, a second-time physical examination is recommended after a few hours of observation. Low-risk cases (BRUE) observation is completed by a 12-lead electrocardiogram and, if a respiratory tract infection is suspected, an RSV test during epidemic season and a *Bordetella pertussis* test in case of incomplete immunization. Other laboratory and imaging investigations are not recommended and discharge after two- to four-hour observation is suggested.

#### 3.14.3. Hospital Admission

Hospitalization is indicated in presence of at least one of the following factors: age < 2 months, preterm with postconceptional age < 43 weeks, need for resuscitation maneuvers or unstable clinical conditions at evaluation, recurrent episodes, episodes occurring during sleep, no correlation with feeding, poor family compliance, and family history of SIDS.

Hospital admission is directed to the identification of underlying causes (i.e., gastroesophageal reflux or other gastrointestinal conditions, metabolic defects, infections, cardiovascular or neurological conditions, abuse). Pulse oximetry monitoring should be prolonged for a minimum of 24 h; meanwhile, first-line laboratory testing should include blood gas analysis with glycemia within the first four hours from the event, whole blood count, electrolytes, C-reactive protein, and urinalysis. Afterwards, a tailored work up should be based on physical examinations and history.

GERD may manifest as ALTE. According to the North American Society for Pediatric Gastroenterology, Hepatology, and Nutrition (NASPGHAN) and the European Society for Pediatric Gastroenterology, Hepatology, and Nutrition (ESPGHAN) definition of GERD, only troublesome regurgitations or gastroesophageal and/or extraesophageal complications of GER require further investigations [13]. Notably, endoscopy is the gold standard for GERD with esophageal symptoms, while pH-impedance is preferred when extra-esophageal symptoms occur or in order to correlate the persistence of symptoms with acid and non-acid GER episodes. If warning signs are not present, neither imaging work up nor pump inhibitor trial are recommended. A trial with alginate for two-to-four weeks may be considered in breastfed infants with persistent regurgitation, when conservative measures are ineffective.

Accidental traumas or abuse must be taken in close consideration in infants with ALTE. Abuse should be suspected especially when anamnestic data reveal incongruences, the family has known social issues or an adult with mental illness or substance abuse lives with the baby. Shaken baby syndrome must be excluded in these cases [14].

Infectious agents account for more than 10% of cases of ALTE [15,16]. When positive history for respiratory symptoms (i.e., nasal congestions, cough, gasping, respiratory distress) is found, a rapid test for RSV is recommended during epidemic season. In the case of paroxysmal cough in infants with incomplete immunization for *Bordetella pertussis*, polymerase chain reaction for pertussis detection from nasopharyngeal swab is suggested [17]. Severe bacterial infections such as pneumonia, meningitis and sepsis are less common and accompanied by unstable conditions at first evaluation.

Respiratory obstruction during sleep is another cause of concern for parents, and it is relatively common in neonates and preterm infants. Once infectious diseases are excluded and suspicion for obstructive sleep apnea syndrome is suspected, a polysomnography is indicated [18].

Neurological conditions may present with BRUE or ALTE. Breath holding spells are benign paroxysmal episodes commonly seen below one year of age and triggered by painful or stressful events. Among neurological diseases, seizures are the most frequent symptoms related with ALTE [3]. However, neurological consultation, EEG recording or neuroimaging were found to have a low sensitivity in a first episode of ALTE without other risk factors [2]. Moreover, seizures may occur as a consequence of hypoxia in prolonged apnea. Thus, it is recommended to complete neurological work-up only in case of recurrence, or when history or physical examination are highly suspicious [19]. Less frequently, a central nervous system infection or malformation, intracranial hypertension, or neurometabolic conditions may mimic an ALTE.

In less than 5% of cases, ALTE hides a metabolic disorder [3]. Signs and symptoms are often insidious and the patient may be asymptomatic at first assessment. In case of recurrent episodes, family history of SIDS, history of failure to thrive or vomiting with neurological symptoms, blood gas analysis with serum lactate and bicarbonate assessment within four hours from episode onset, blood ammonia, glycemia, ketonemia, and urinalysis are suggested as first line laboratory exams. Afterwards, a specialist consultation should be performed and, if needed, a second-line work up including organic acids and plasma amino acids could drive physicians in differential diagnosis [20].

Heart diseases and rhythm anomalies like prolongation of QT interval or Wolff–Parkinson–White syndrome may be a rare cause of ALTE [21]. A 12-lead ECG is recommended as a first level screening in patients with BRUE or ALTE. In high-risk cases, cardiorespiratory monitoring should be prolonged during the first 24 h of hospitalization. Further investigations will be indicated by the specialists in case of suspected heart disease.

When no other conditions are identified, a diagnosis of idiopathic ALTE may be made.

#### 3.14.4. Prognosis

At discharge, clinicians should reassure parents, educating them about BRUE and ALTE, providing cardiopulmonary resuscitation training and psychological support when needed, with the help of primary care services. Depending on the cause of ALTE, a multidisciplinary follow-up with the central role of the general pediatrician is recommended. This will help unveil underlying causes and monitor the clinical conditions. There is high variability in the literature concerning the risk of recurrence or death in ALTE [22]. Victims of abuse, patients requiring resuscitation maneuvers or those with underlying neurological conditions are at higher risk of death.

Cardiorespiratory home monitoring with data recorder and adjustable alarms is suggested in high-risk cases (i.e., family history of SIDS, recurrent episodes of ALTE, preterm with postconceptional age < 43 weeks). A duration of twelve weeks or until the 43rd week of postconceptional age is suggested for monitoring, unless different specialist indication is present.

## 4. Discussion

The management of BRUE and ALTE has always been troublesome for clinicians and of high concern for caregivers. So far, the guidelines already published did not provide sufficient level of evidence for several indications. The selection of an appropriateness method for medical choices and procedures expected for BRUE and ALTE is crucial, as it allows combining current evidence with medical experience, with the purpose of developing a uniform shared protocol. As for the existing evidence, we referred to the American and Italian Societies of Pediatrics guidelines, updated in 2016 and 2017, respectively. Results derived from panel meeting and discussion showed points of disagreement with different opinion emerged, and the need for focusing education on some areas.

When considering the prevention of unexpected events, inadequate rate of agreement was achieved on the advice against pillows in the baby’s cradle. Given the risk of suffocation and entrapment with soft objects during sleep [23,24], it is important to discourage new parents from the use of pillows and to improve primary care pediatricians’ skills in the field of SIDS prevention with online courses.

The indications for ER evaluation and hospitalization defined by the Italian guidelines were strongly confirmed by our panel. In comparison with the indications by Piumelli et al. [3], the age limit for hospitalization was raised up to two months, so as to include the entire age group at higher risk for ALTE. However, the need for a prompt evaluation of low-risk patients with milder episodes (i.e., first episode of brief duration in infants older than two months irrespective of other risk factors, or irregular breathing lasted more than one minute without color or muscle tone change in a patient below two months of age) reached an indeterminate agreement. A reasonable explanation may be the difficulty to determine the precise nature of each BRUE in everyday clinical practice. In line with the key action statements by Tieder et al. [2], it is worthwhile to conduct an educational campaign for new parents in local settings, and to guarantee a primary care support with in-person and telemedicine visits for all those cases not requiring urgent hospital admission.

Local ER management of low-risk patients showed discordant opinions among panelists, with the majority disagreeing from the existing guidelines indications. In particular, high appropriateness levels were observed for routine blood tests, chest X-ray and neurological consultation in patients with BRUE, in contrast with the American recommendations. After meeting debate, final decision for protocol drafting was to maintain a non-invasive approach and to avoid further stressing, worthless and counterproductive tests, unless differently suggested by history and physical examination. An additional point of disagreement concerned the duration of ER observation. The threshold of two- to four-hour suggested by Tieder et al. [2] was ranked appropriate for the 54.5% of panelists, but inappropriate for the 27.3% of them. The four-hour threshold was then kept for guidance in our local protocol, since this threshold may be substantially influenced by several factors, such as family compliance, caregivers’ training, workload, and local ER organization.

With regard to the hospital management of suspected gastroesophageal, metabolic and sleep related conditions, the survey and panel meeting conclusions were consistent with guidelines recommendations [3,13,18,20]. Analogue results were obtained for the indications of first step laboratory diagnostic work up. On the contrary, the infectious diseases diagnostic approach obtained an indeterminate level of agreement, in particular for pertussis and RSV testing, unlike the American guidelines for BRUE [2] suggesting that a polymerase chain reaction test for pertussis and RSV should be always performed. Our panel highlighted the need to implement medical education on association between respiratory infections and BRUE in local settings. 

The pulse oximetry monitoring of hospitalized patients can help detecting possible relapses that are reported to occur in over the 30–50% of severe cases [25]. Participants suggested 24 h as the minimum threshold of monitoring, as acute relapses are mostly reported in this time interval. The extension of cardiorespiratory monitoring at home after discharge has been employed for years in specific at-risk categories. The conclusion of our survey showed a large consensus for the selection of such categories, i.e., patients with recurrent ALTE, premature with a postconceptional age lower than 43 weeks, and patients with a positive family history of SIDS. The Italian guidelines recommended home monitoring for severe or recurrent ALTE and for symptomatic preterm infants, in agreement with previous studies [26,27,28]. Even when correlation between SIDS and ALTE has been ruled out, home monitoring may help the diagnostic pathway and reassure parents, especially if they have already experienced a SIDS. For this reason, our panel focused also on ALTE cases with a past family history of SIDS, so as to activate a local program of prevention and follow-up. However, the adequate time length of home monitoring is still controversial. Although the achievement of the 43rd postconceptional week of age in preterm has been reported as a safe cutoff for the reduction of extreme events [29,30], the remaining at risk-population is not homogeneous and a clear threshold is difficult to be defined. Piumelli et al. proposed a six weeks monitoring, extendible up to 12 weeks for symptomatic patients [3]. Our expert panel did not find a final consensus and the prolongation of cardiorespiratory recording for 12 weeks in the general at-risk population reached a 54.5% rate of appropriateness. Such results prove that further studies aimed at detecting valid biomarkers of severe ALTE or SIDS are required, as they and would be of exceptional help in everyday clinical practice.

Our study has some limitations. The number of participants was limited and they worked in the same region of the same country. The application of similar approach to other regions and countries may be of great interest for a comparative assessment. Secondly, the questionnaire encompasses many critical scenarios for BRUE and ALTE clinical management, but some aspect has been left out in order to maintain a good level of manageability for participants, and focus on the critical keys. However, to our knowledge, this is one of the few studies addressing RAND appropriateness method to pediatric population. It represents a multidisciplinary effort for supporting decision making in a field lacking of robust evidence-based studies, meantime providing shared indications suitable for our local setting.

## 5. Conclusions

By combining previous recommendations with expert opinion, the RAND/UCLA appropriateness method permitted to drive pediatricians to reasoned and informed decisions in terms of evaluation, treatment, and follow-up of BRUE and ALTE, by reducing inappropriate exams and hospitalization and highlighting priorities for educational interventions.

## Figures and Tables

**Table 1:** Definition of Sudden Infant Death Syndrome (SIDS), Brief Resolved Unexpected Event (BRUE) and Apparent Life-Threatening Event (ALTE).

Definition	Definition
**SIDS**	Sudden, unexpected death before 12 months of age occurring in a previously healthy infant, in which the cause remains unknown despite thorough investigations (including an appropriate autopsy, death scene investigation, and analysis of the clinical history).
**BRUE**	Sudden, brief (<1 min) episode without other explainable cause occurring in an infant younger than 12 months of age characterized by ≥1 among cyanosis or pallor, absent/ decreased/ or irregular breathing, marked change in tone, altered level of responsiveness.
**ALTE**	Episode that is frightening to the observer, characterized by ≥1 among apnoea (central or occasionally obstructive), color change, marked change in muscle tone, choking or gagging.

**Table 2:** History collection in patients with suspected BRUE/ALTE.

History	Information
**Family history**	Episodes of SIDS or sudden death in family members before age 35 years Episodes of ALTE/BRUE Cardiac diseases (i.e., long QT syndrome, arrhythmia) Inborn error of metabolism or genetic disease Developmental delay Allergies Epilepsy (i.e., infantile spasms) Malformations
**Past history**	Pre-/perinatal history Prematurity and neonatal history Past medical history Feeding problems, reflux Growth Neurocognitive development, behavior Previous episodes/BRUE Respiratory problems (in sleep and wakefulness) Past injuries Previous hospitalization Immunization Medications
**Recent history**	General conditions in the previous 48 h (i.e., illness, signs and symptoms, feeding problems, vaccinations) Injuries, falls, unexplained bruising Drugs New food introduction Sleep pattern alterations, sleep deprivation
**History of the event**	Full description Duration Person that reported the event Witnesses of the event (and level of reliability)
**Circumstances of the event**	Location Position of the baby (i.e., prone, supine, sitting) If awake: ask for sounds, breathing abnormalities, vomit, regurgitation If asleep: ask for cough, vomit, rigidity or cry before the episode During feeding (or time since last feeding) During the bath Ambiental risk factors: smoke, CO, temperature, clothing, blankets, close objects, accidental events
**Appearance of the baby during the event**	Skin color and lips color Muscle tone Consciousness Limb movements, other movements Skin temperature Sweating Respiratory distress Apnea Bleeding
**End of the event and action**	Duration (</≥1 min) Baby aspect before resolution Spontaneous resolution or after intervention Gradual or rapid resolution Time from beginning to first intervention Time from first intervention to return to normality Need for vigorous interventions or resuscitation maneuvers Intervention provided by caregiver or healthcare personnel
**Socio-environmental history**	Family structure Home condition Recent changes, stressful conditions, or conflicts Drugs/toxic substances exposure Social services assistance Psychiatric problems Adults with history of substance abuse
**Considerations for possible child abuse**	Social services assistance Incongruences between the descriptions and child’s developmental stage Infant blamed for bad behavior Changing versions of the history Unexplained bruises

**Table 3:** Physical examination of a patient with a suspected BRUE/ALTE.

**General conditions and vital signs**	Auxological parameters General aspect (color, perfusion/ capillar refill, crying, hydration) Respiratory function Cardiac function, pulse, pressure Vital signs monitoring
**Physical examination**	Eyes (i.e., extrinsic ocular motility, pupillary response, conjunctiva, fundoscopy) Ears and pharynx (i.e., nasal congestion and secretions, blood in nostrils or oral cavity, evidence of injuries or obstructions) Chest (i.e., ribs fractures, asymmetry) Abdomen (i.e., organomegaly, masses, abdominal distension) Genitals Skin (i.e., bruises or other lesions) Dysmorphisms (i.e., especially craniofacial) Skeletal muscle (i.e., neck mobility, other joints, lesions, deformities, fractures, muscle tone, and strength) Nervous system (i.e., alertness, responsiveness, response to visual and sound stimuli, tendon reflexes, meningeal symptoms, asymmetries of the movements, anterior fontanelle)

## Data Availability

Data is contained within the article.

## Supplementary Materials

The following are available online at https://www.mdpi.com/2075-1729/11/2/171/s1.
