# Supplementary material for: Management of Infants with Brief Resolved Unexplained Events (BRUE) and Apparent Life-Threatening Events (ALTE): A RAND/UCLA Appropriateness Approach
Source: Life (Basel). 2021 Feb 22;11(2):171. doi: 10.3390/life11020171 (PMC7926945; doi:10.3390/life11020171)
Supplement: Supplementary file 1 [file life-11-00171-s001.pdf]

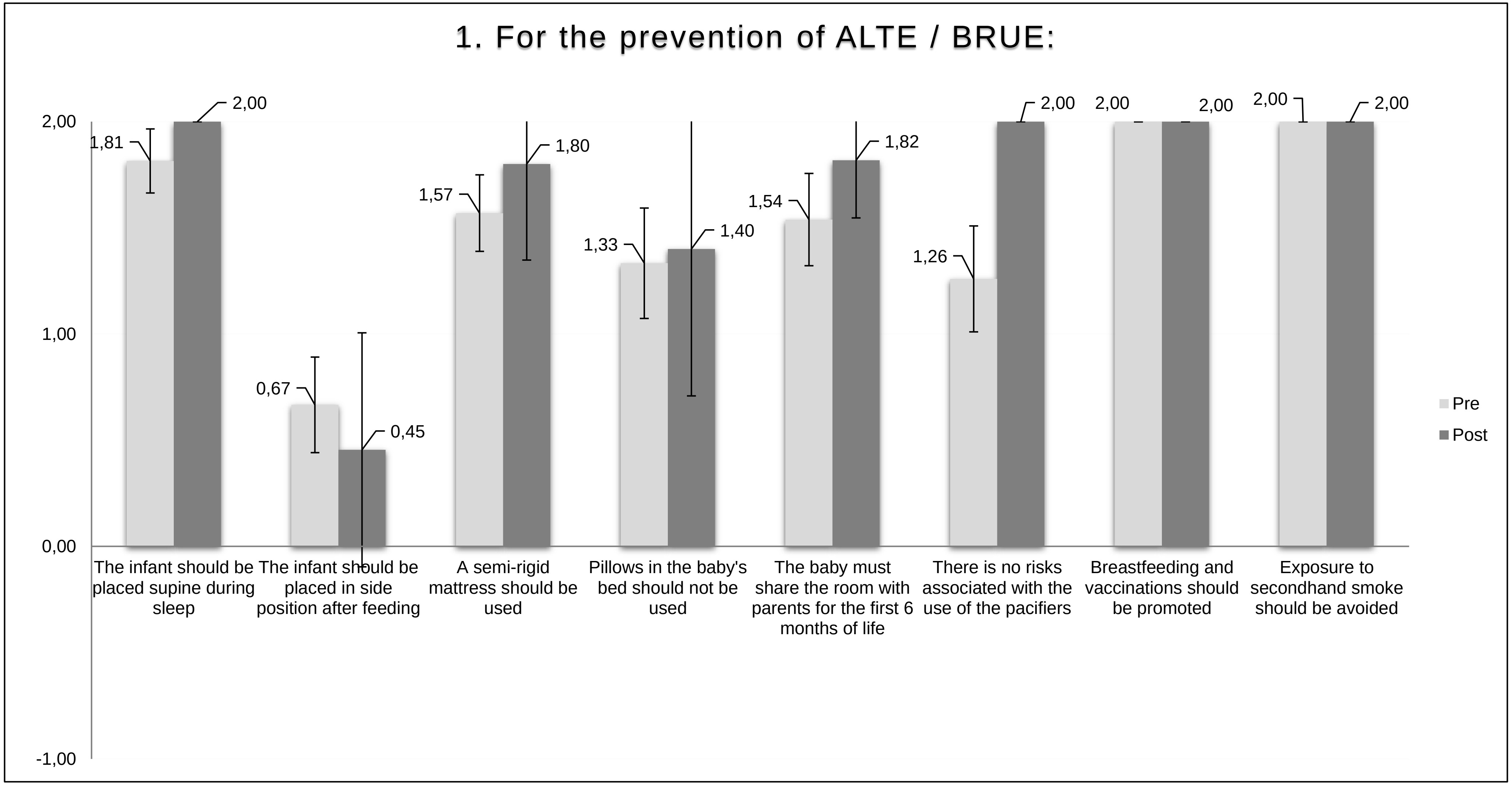

1. For the prevention of ALTE / BRUE:
2,00
1,00
0,00
-1,00
1,81
2,00
0,67
0,45
1,57
1,80
1,33
1,40
1,54
1,82
1,26
2,00
2,00
2,00
2,00
2,00
Pre
Post
The infant should be placed supine during sleep
The infant should be placed in side position after feeding
A semi-rigid mattress should be used
Pillows in the baby's bed should not be used
The baby must share the room with parents for the first 6 months of life
There is no risks associated with the use of the pacifiers
Breastfeeding and vaccinations should be promoted
Exposure to secondhand smoke should be avoided

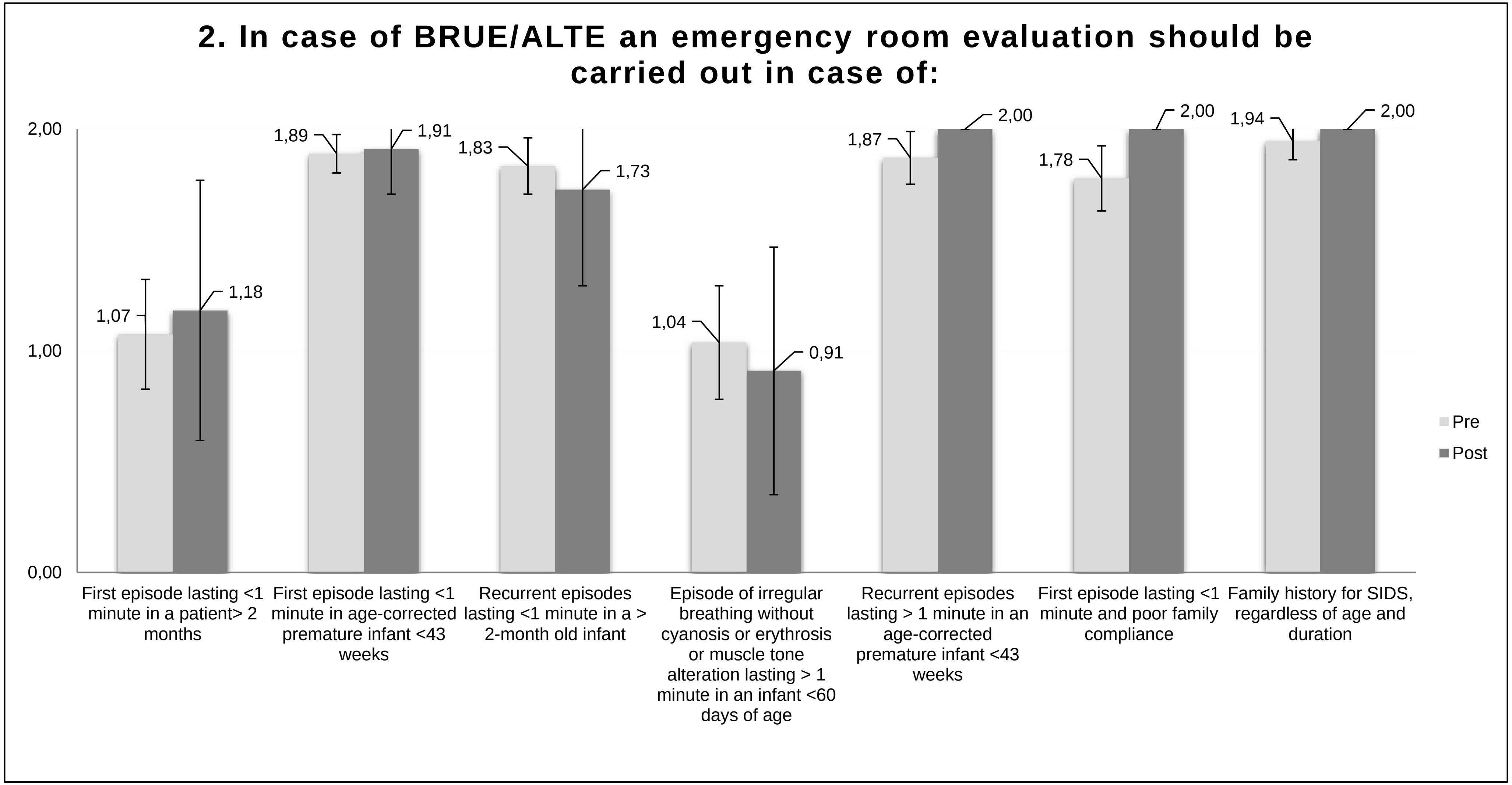
2. In case of BRUE/ALTE an emergency room evaluation should be carried out in case of:
2,00
1,00
0,00
1,07
1,18
1,89
1,91
1,83
1,73
1,04
0,91
1,87
2,00
1,78
2,00
1,94
2,00
Pre
Post
First episode lasting <1 minute in a patient> 2 months
First episode lasting <1 minute in age-corrected premature infant <43 weeks
Recurrent episodes lasting <1 minute in a > 2-month old infant
Episode of irregular breathing without cyanosis or erythrosis or muscle tone alteration lasting > 1 minute in an infant <60 days of age
Recurrent episodes lasting > 1 minute in an age-corrected premature infant <43 weeks
First episode lasting <1 minute and poor family compliance
Family history for SIDS, regardless of age and duration

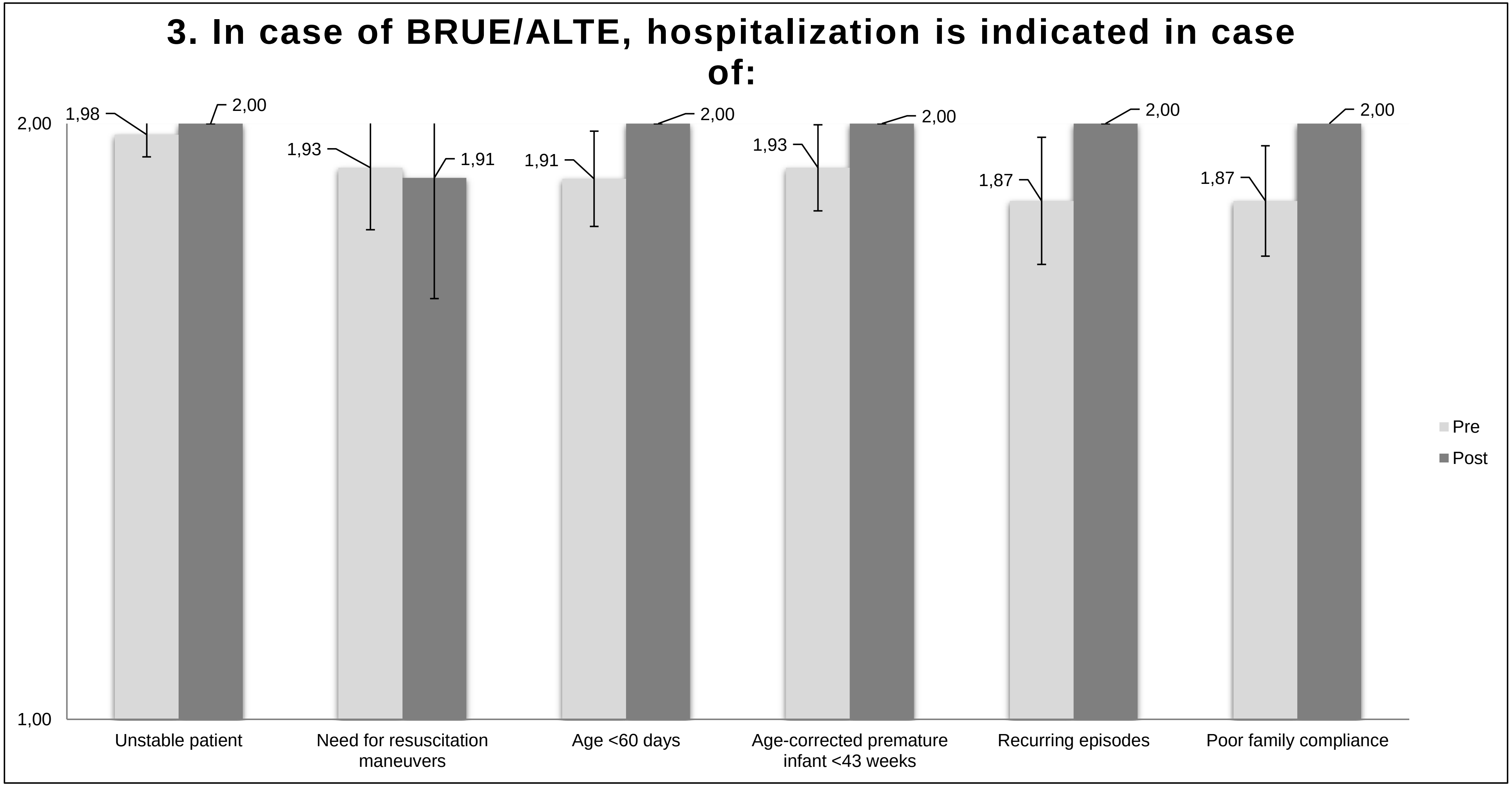

3. In case of BRUE/ALTE, hospitalization is indicated in case of:
2,00
1,00
1,98
2,00
1,93
1,91
1,91
2,00
1,93
2,00
1,87
2,00
1,87
2,00
Unstable patient
Need for resuscitation maneuvers
Age <60 days
Age-corrected premature infant <43 weeks
Recurring episodes
Poor family compliance
Pre
Post

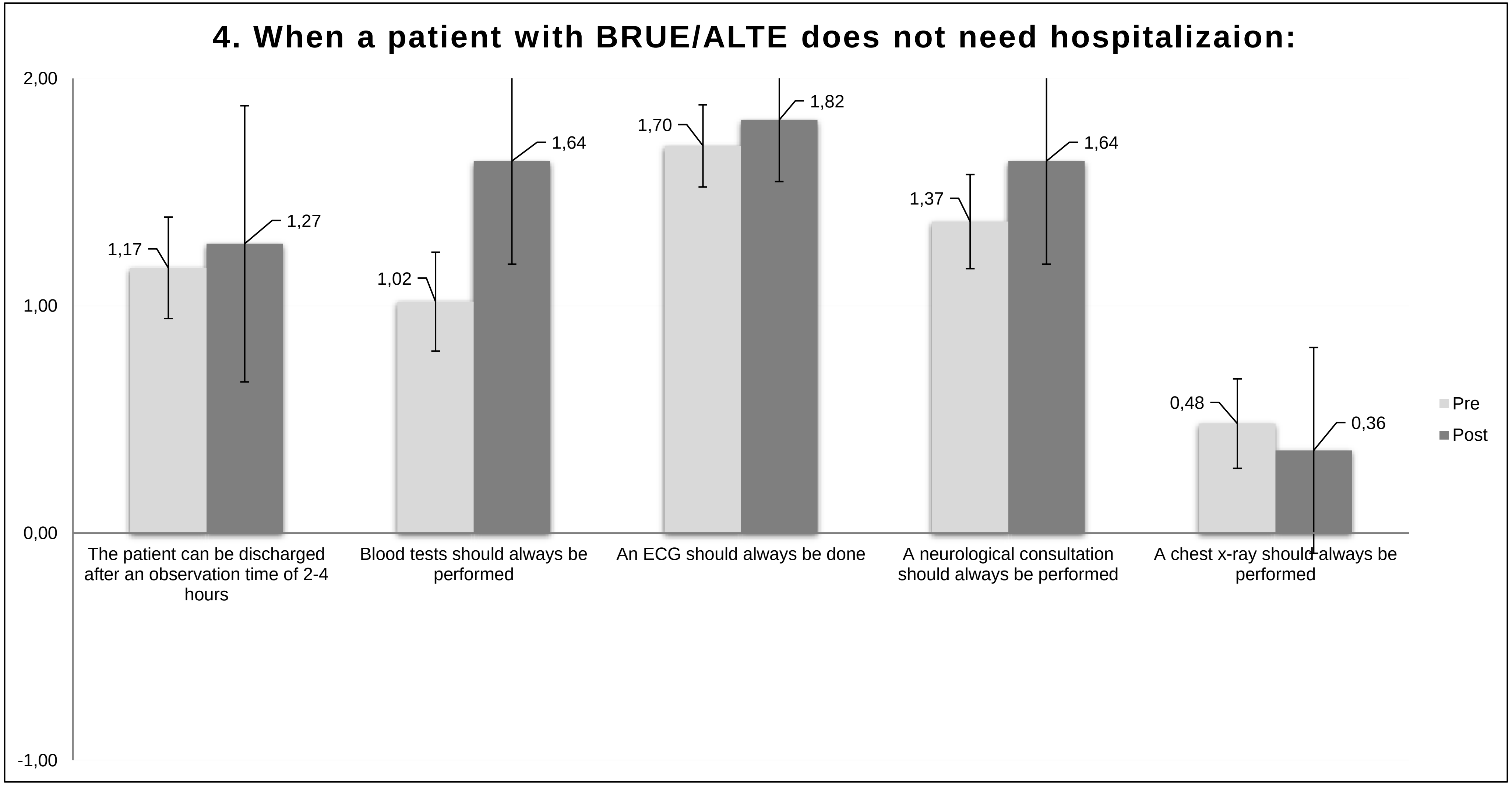
4. When a patient with BRUE/ALTE does not need hospitalizaion:
2,00
1,00
0,00
-1,00
1,17
1,27
1,02
1,64
1,70
1,82
1,37
1,64
0,48
0,36
Pre
Post
The patient can be discharged after an observation time of 2-4 hours
Blood tests should always be performed
An ECG should always be done
A neurological consultation should always be performed
A chest x-ray should always be performed

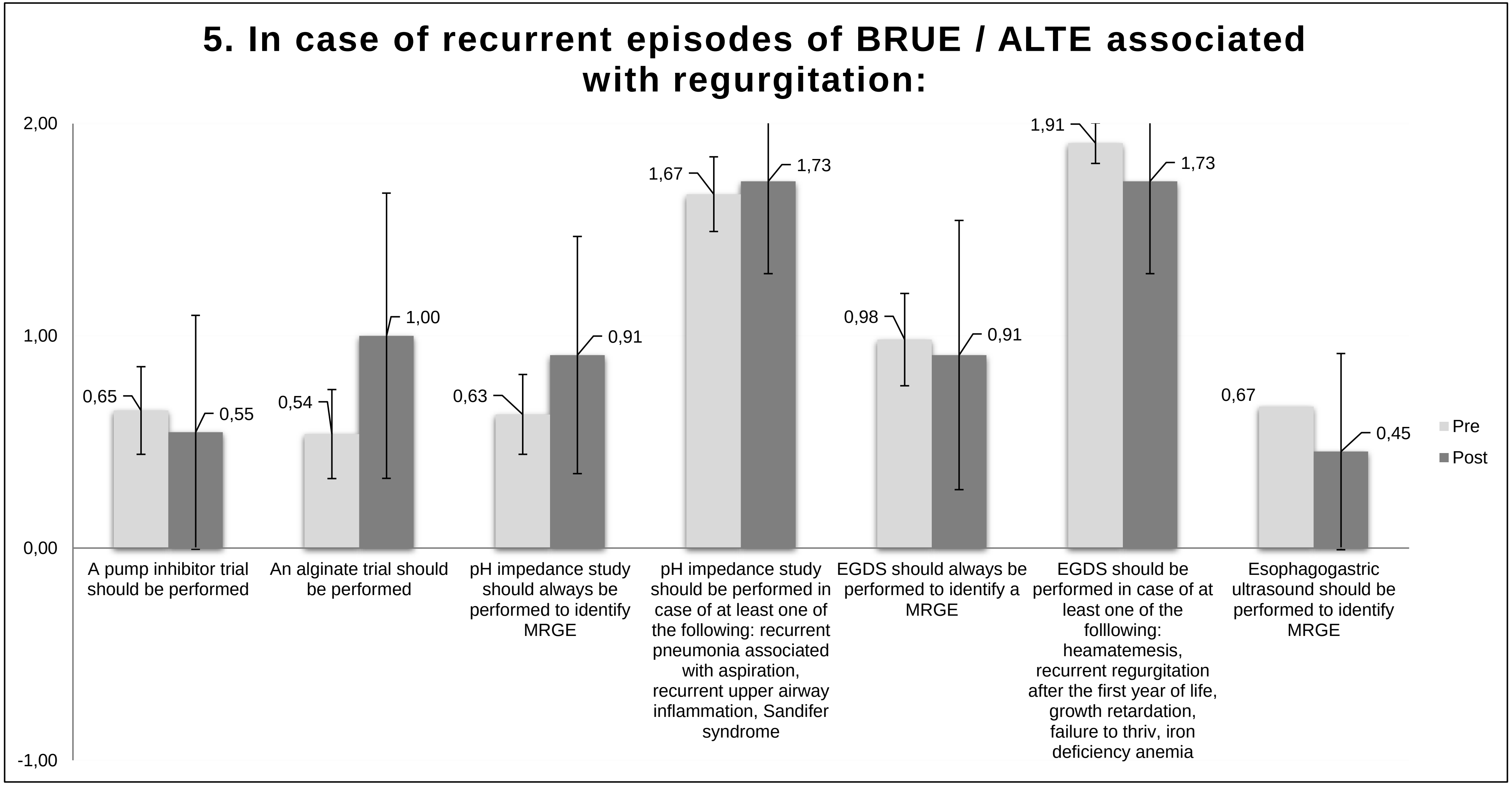
5. In case of recurrent episodes of BRUE / ALTE associated with regurgitation:
2,00
1,00
0,00
-1,00
0,65
0,55
0,54
1,00
0,63
0,91
1,67
1,73
0,98
0,91
1,91
1,73
0,67
0,45
Pre
Post
A pump inhibitor trial should be performed
An alginate trial should be performed
pH impedance study should always be performed to identify MRGE
pH impedance study should be performed in case of at least one of the following: recurrent pneumonia associated with aspiration, recurrent upper airway inflammation, Sandifer syndrome
EGDS should always be performed to identify a MRGE
EGDS should be performed in case of at least one of the folllowing: heamatemesis, recurrent regurgitation after the first year of life, growth retardation, failure to thriv, iron deficiency anemia
Esophagogastric ultrasound should be performed to identify MRGE

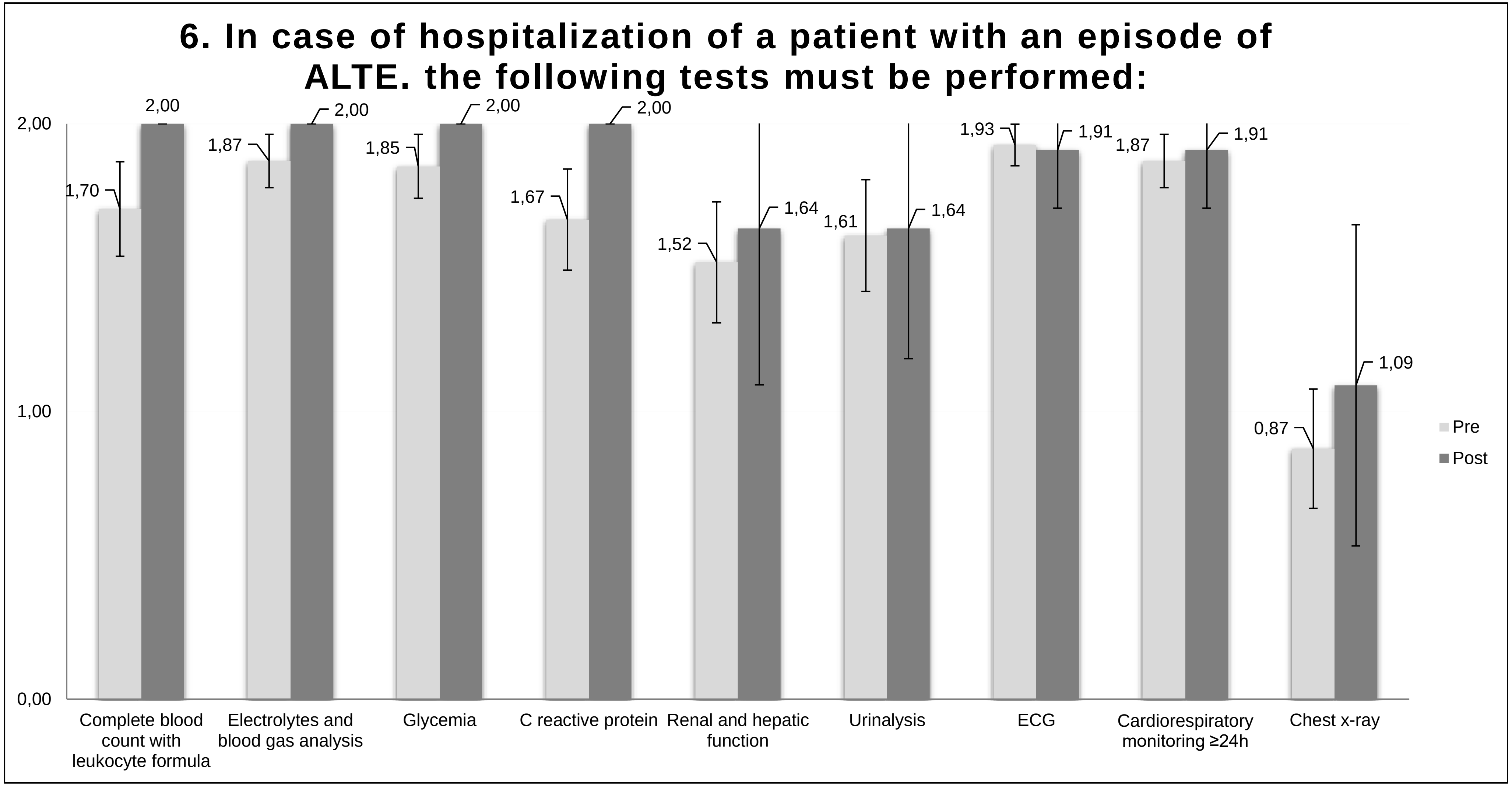
6. In case of hospitalization of a patient with an episode of ALTE. the following tests must be performed:
2,00
1,00
0,00
1,70
2,00
1,87
2,00
1,85
2,00
1,67
2,00
1,52
1,64
1,61
1,64
1,93
1,91
1,87
1,91
0,87
1,09
Complete blood count with leukocyte formula
Electrolytes and blood gas analysis
Glycemia
C reactive protein
Renal and hepatic function
Urinalysis
ECG
Cardiorespiratory monitoring ≥24h
Chest x-ray
Pre
Post

7. In the event of a BRUE / ALTE episode, tests for infectious disease should be performed:

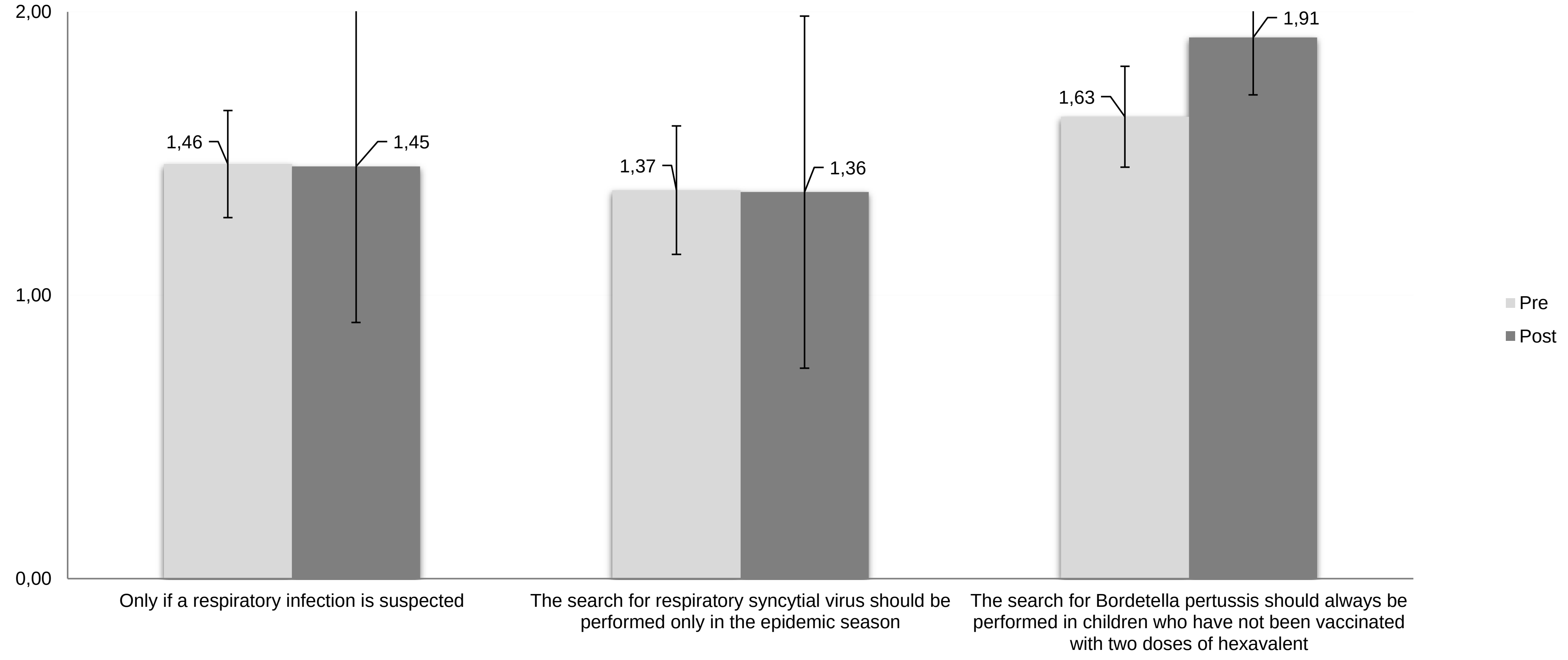

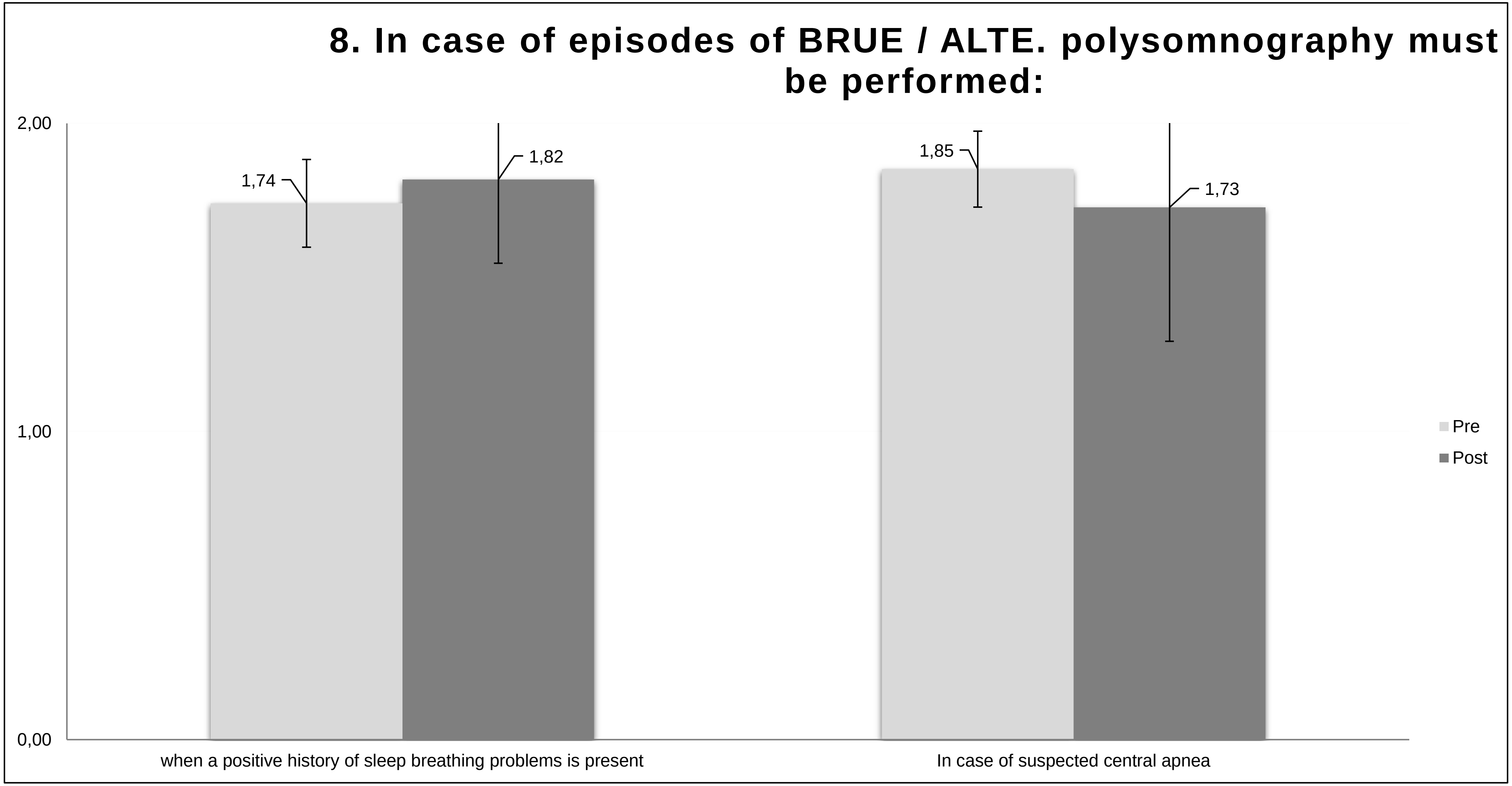
8. In case of episodes of BRUE / ALTE. polysomnography must be performed:
2,00
1,00
0,00
1,74
1,82
1,85
1,73
Pre
Post
when a positive history of sleep breathing problems is present
In case of suspected central apnea

9. A metabolic disease should be suspected when BRUE / ALTE episodes are associated with:

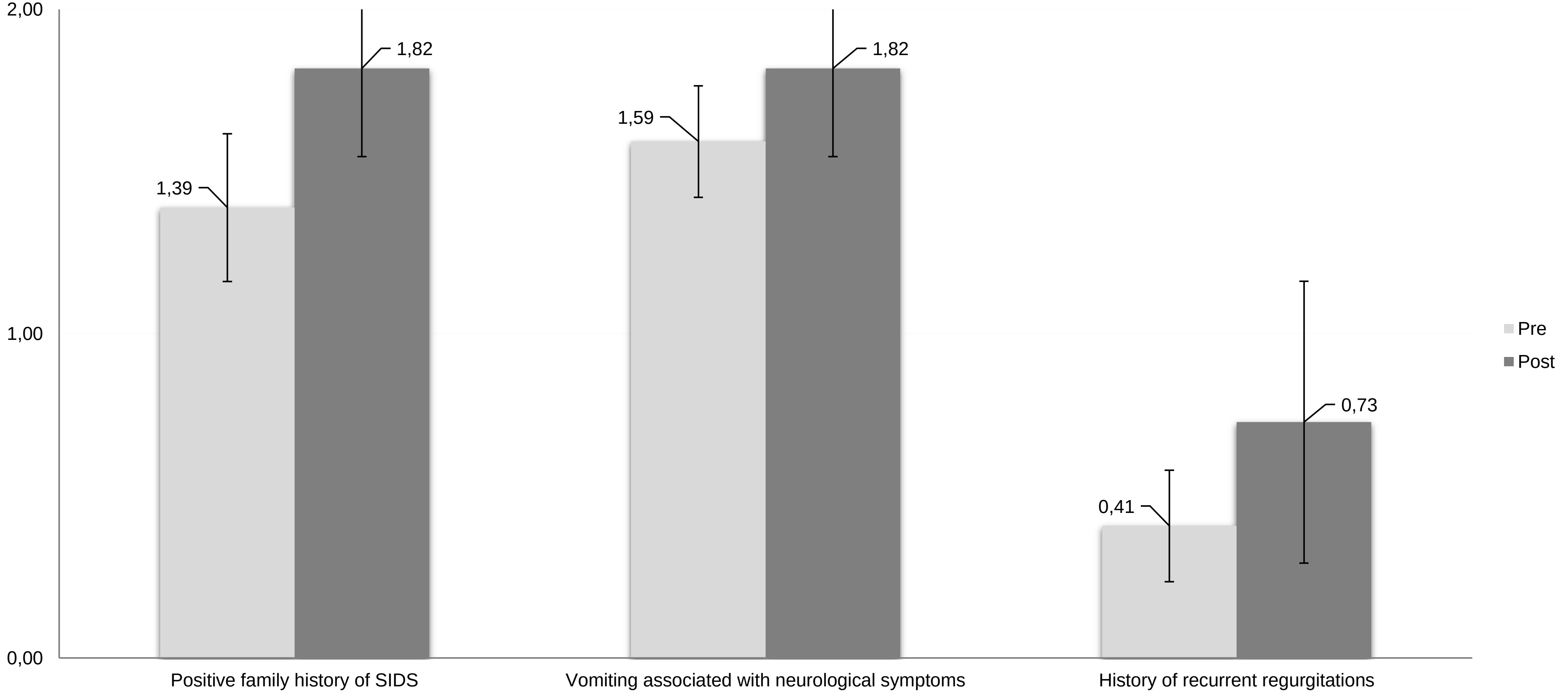

10. In case of ALTE with suspected metabolic disease it is recommended to perform:

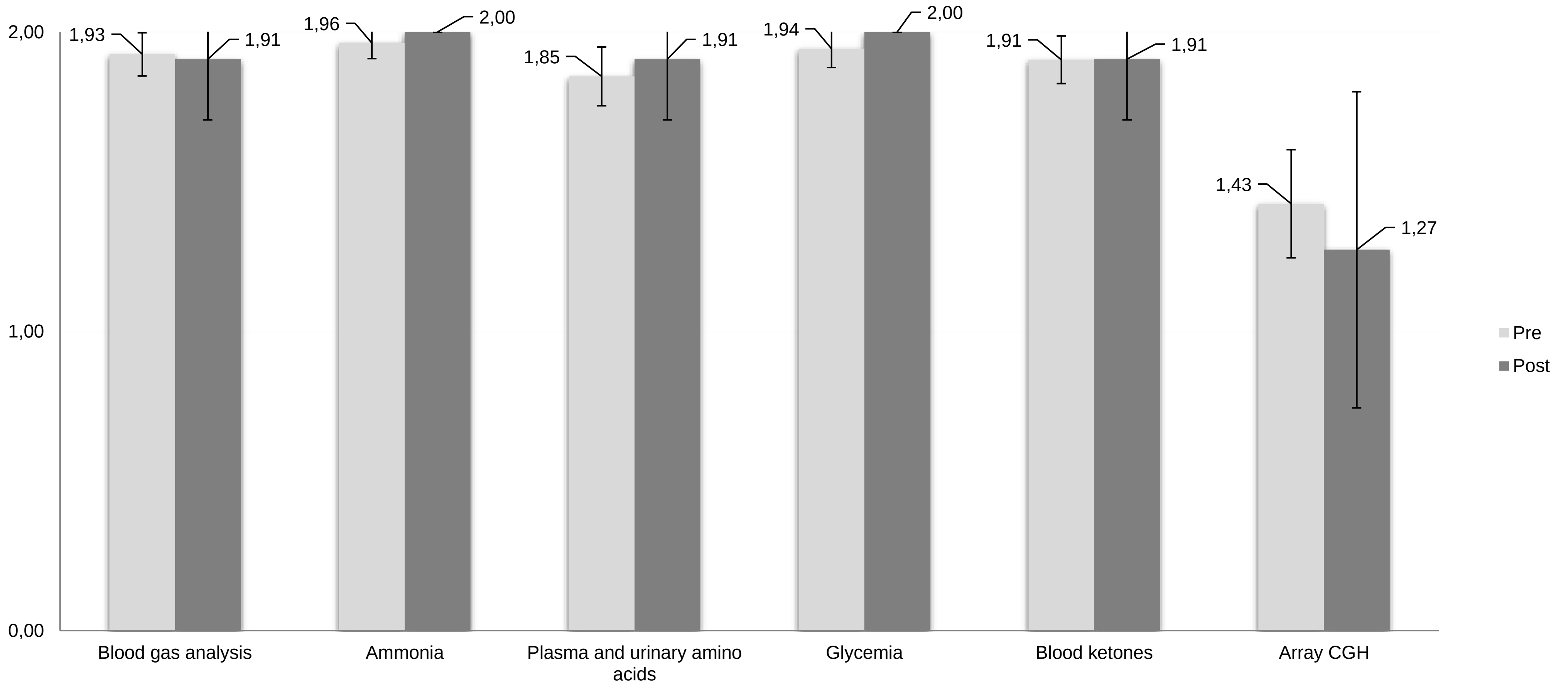

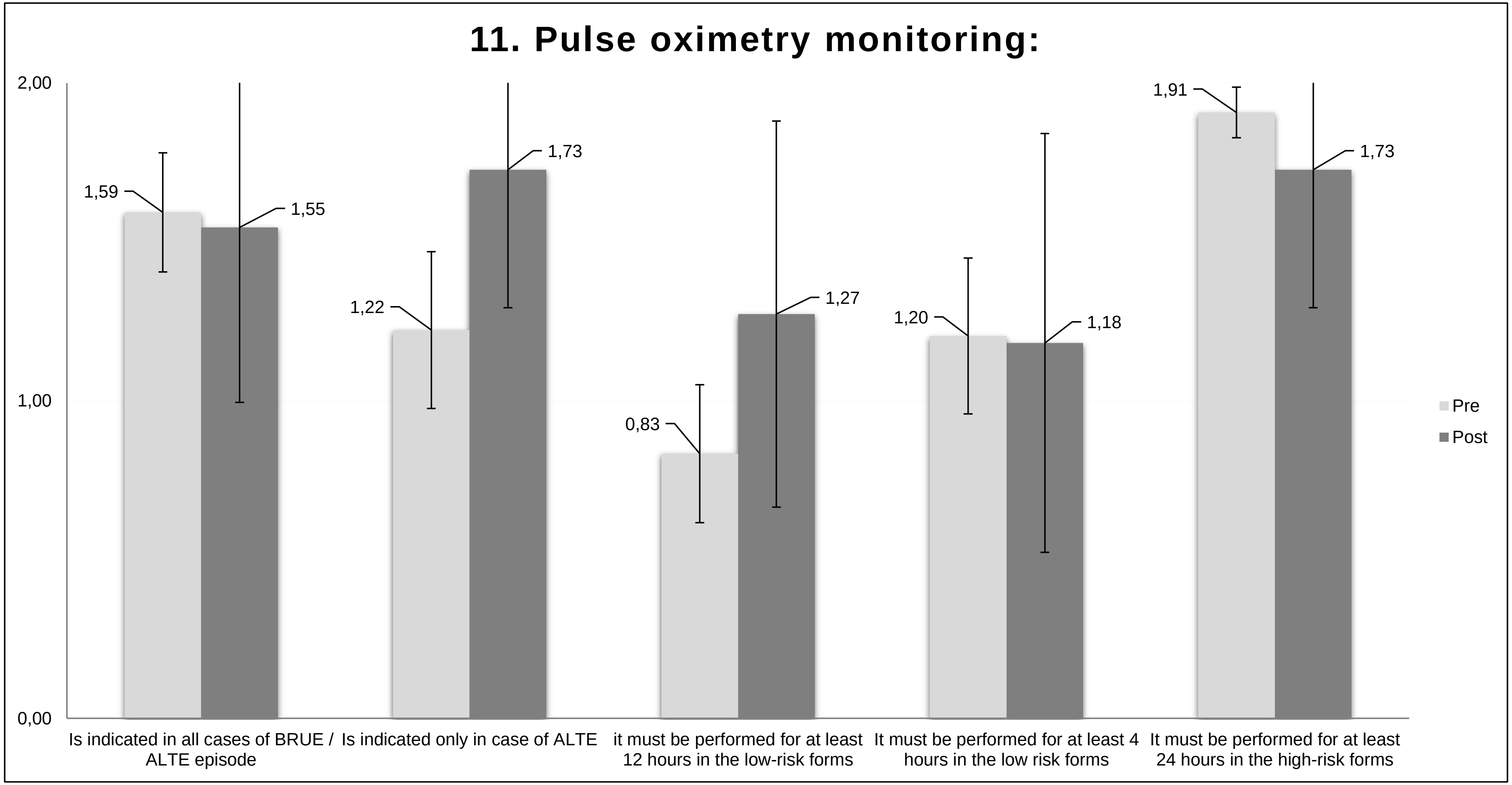
11. Pulse oximetry monitoring:
2,00
1,00
0,00
1,59
1,55
1,22
1,73
0,83
1,27
1,20
1,18
1,91
1,73
Pre
Post
Is indicated in all cases of BRUE / ALTE episode
Is indicated only in case of ALTE
it must be performed for at least 12 hours in the low-risk forms
It must be performed for at least 4 hours in the low risk forms
It must be performed for at least 24 hours in the high-risk forms

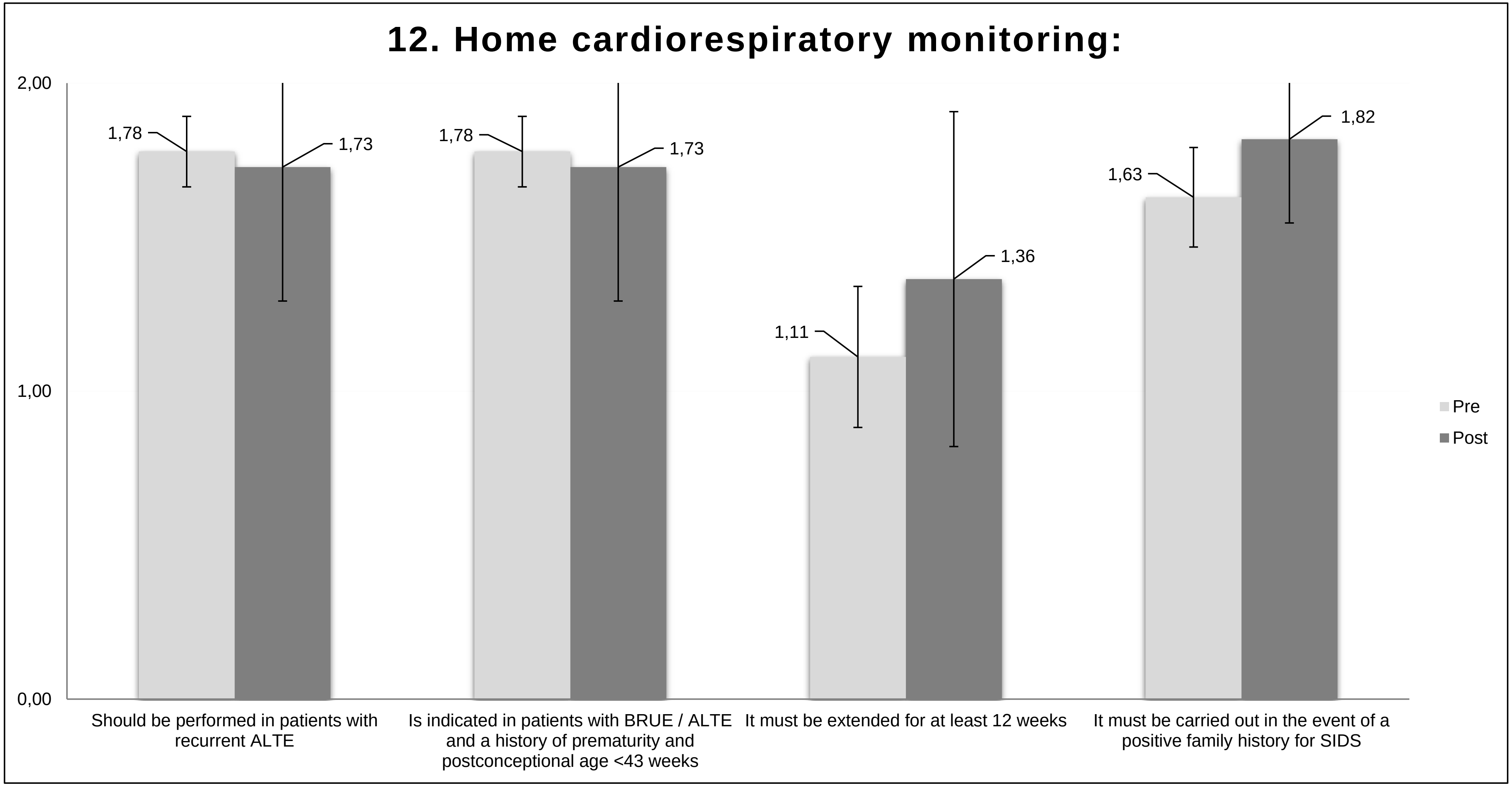
12. Home cardiorespiratory monitoring:
2,00
1,00
0,00
1,78
1,73
1,78
1,73
1,11
1,36
1,63
1,82
Pre
Post
Should be performed in patients with recurrent ALTE
Is indicated in patients with BRUE / ALTE and a history of prematurity and postconceptional age <43 weeks
It must be extended for at least 12 weeks
It must be carried out in the event of a positive family history for SIDS
